# Supplementary material for: Heterozygous NPR2 Variants in Idiopathic Short Stature
Source: Genes (Basel). 2022 Jun 15;13(6):1065. doi: 10.3390/genes13061065 (PMC9222219; doi:10.3390/genes13061065)
Supplement: Supplementary file 1 [file genes-13-01065-s001.zip › genes-1748897-supplementary.pdf]

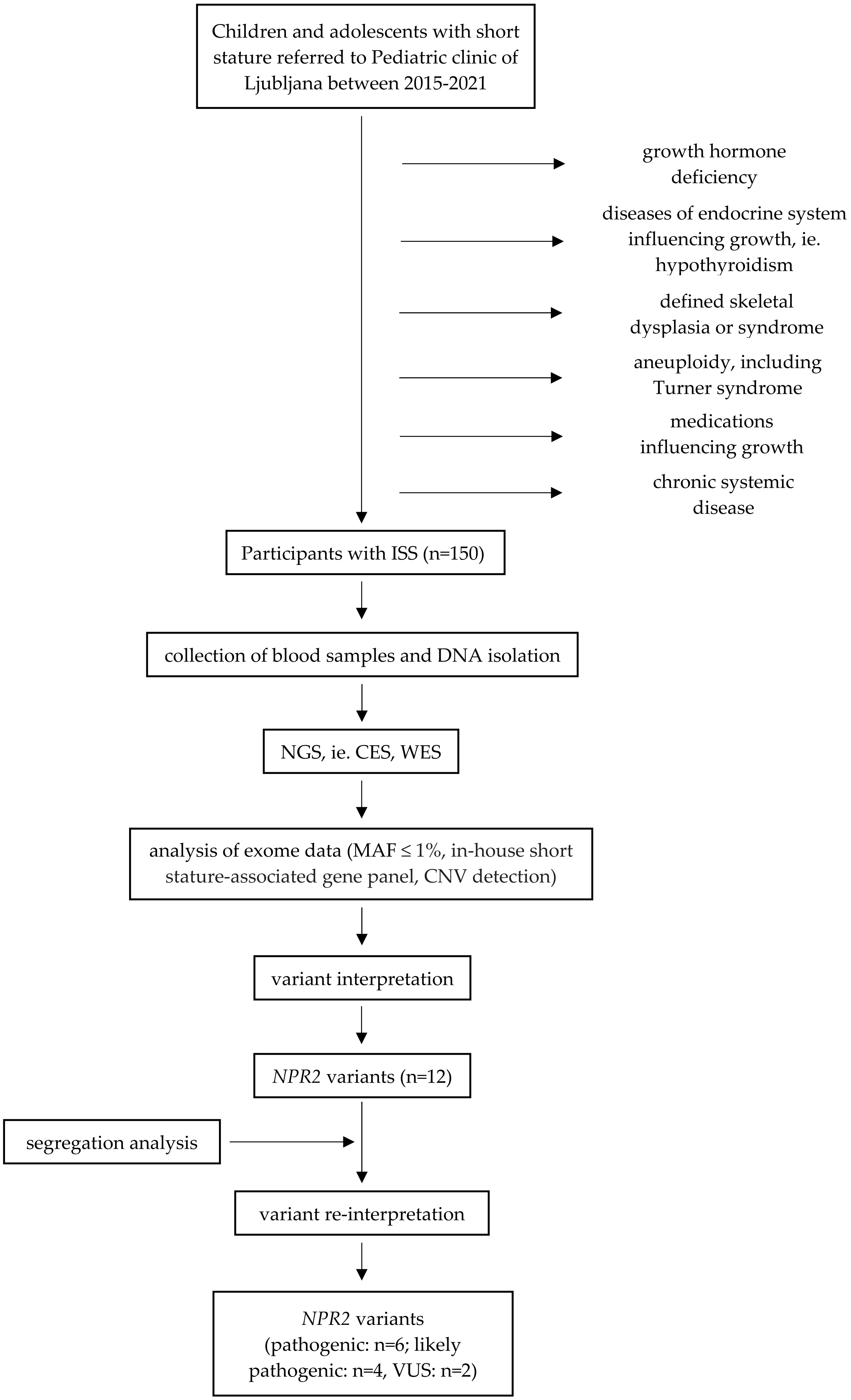

Supplement S1: Study flowchart. ISS – idiopathic short stature, NGS – Next-Generation Sequencing, CES – Clinical Exome Sequencing, WES – Whole Exome Sequencing, MAF – minor allele frequency, VUS – variant of uncertain significance
